# Role of the right anterior insula for the emergence of flow—A combined task-based fMRI activation and connectivity study

**DOI:** 10.3389/fnhum.2022.1067968

**Published:** 2022-12-08

**Authors:** Martin Ulrich, Filip Niemann, Georg Grön

**Affiliations:** ^1^Section Neuropsychology and Functional Imaging, Department of Psychiatry, Ulm University, Ulm, Germany; ^2^Cognition, Aging, and Brain Stimulation Lab, Department of Neurology, University Medicine Greifswald, Greifswald, Germany

**Keywords:** flow experience, salience, anterior insula, putamen, functional connectivity, functional magnetic resonance imaging, intrinsic motivation

## Abstract

The emergence of flow is a situation of high salience because externally oriented attention on the task and access to resources for goal-directed behavior are enhanced, while internally oriented or self-related cognition is decreased. The right anterior insula has been reported as a causal out-flow hub of the salience resting-state network, orchestrating the engagement of the central executive network (CEN) and the disengagement of the default-mode network (DMN) during a functional challenge. In the present study, we employed a combined task-based activation and connectivity analysis to investigate the role of the right anterior insula during the emergence of flow. A sample of 41 healthy male subjects was confronted with a functional challenge that permitted the emergence of flow during BOLD-based functional magnetic resonance imaging. Comparing connectivity changes in the right anterior insula during the flow condition against connectivity changes associated with control conditions of boredom and overload, relatively increased couplings were observed with the left and right dorsolateral prefrontal cortex. Activation data for these regions did, however, not show the flow-typical inverted U-shaped (invU) response pattern. Relatively decreased functional couplings encompassed ventral aspects of the striatum, but neither the amygdala nor the medial prefrontal cortex (MPFC). For the ventral striatum, activation data were consistent with the flow-typical U-shaped activation pattern, which supports the notion that under the high salience of autotelic situations, the anterior insula is much less positively coupled with the ventral striatum than under boundary conditions of boredom and overload. Taken together, present functional connectivity results were in alignment with the assumed role of the right anterior insula under conditions of different salience. However, this particular region does not appear to mediate the most typical flow-associated activation patterns.

## Introduction

The concept of flow (Csikszentmihalyi, 1975) refers to an activity-associated, subjective experience with the following characteristic features: high albeit almost effortless attention, reduced self-referential processing, sense of control, and the feeling that the activity *per se* is rewarding (Csikszentmihalyi, 2000; Csikszentmihalyi and Nakamura, 2010). This subjective experience of flow can arise during physical and mental tasks that fit with the most important prerequisite: an optimal balance between actual task affordances and individual skills and/or abilities in the context of clear goal settings. Situations, where this prerequisite cannot be met, are unlikely to permit the experience of flow, giving rise to flanking subjective experiences, that is, boredom or overload. The latter experiences, therefore, constitute the boundary conditions against which the emergence of flow is tested because the entire concept of flow is operationally defined relative to these boundary conditions. Particularly the autotelic feature brings the psychological construct flow conceptually into the field of intrinsic motivation, which is opposite to extrinsic motivation denoting the availability of extrinsic rewards (e.g., food and money) as the driving force behind the initiation and maintenance of actions.

In our previous studies on the neural correlates and mechanisms of the flow experience during mental arithmetic (Ulrich et al., 2014; Ulrich et al., 2016a,b; Ulrich et al., 2018b), we have particularly focused on those experiential features that were relatively more absent during flow (self-referential processing and emotional arousal). This relative absence was mirrored by and correlated with reduced neural activation [medial prefrontal cortex (MPFC), amygdala] during flow relative to the boundary conditions. When psychological conditions of boredom, flow, and overload are arranged in that order, this activation pattern can, therefore, be inferred by a quadratic trend test mirroring a U-shape.

Inverting the contrast, we also observed a robust inverted U-shaped (invU) activation pattern of various brain regions. Generally, the interpretation of how this activation pattern may relate to the experience of flow is more ambiguous than for the U-shaped activation, as contributions of the experimental task *per se* (arithmetic calculation) and associated subjective experiences cannot easily be disentangled. Still, some of those brain regions with an invU-shaped activation pattern are less likely to be particularly driven by the mental arithmetic task than other regions, and of those, the right anterior insula with a robust invU-shaped activation pattern increasingly gained our research interest for two reasons. First, particularly the right anterior insula has been reported (Sridharan et al., 2008; Menon and Uddin, 2010; Menon, 2011, 2015) as a causal out-flow hub of the so-called salience resting-state network orchestrating the engagement of the central executive network (CEN) and the disengagement of the default-mode network (DMN) during a functional challenge [for further support of this mechanism, see also work by Goulden et al. (2014), Chand and Dhamala (2016), Krönke et al. (2020) and Shaw et al. (2021)]. Second, the flow condition aligns well with a situation of high salience because externally oriented attention on the task and access to resources for goal-directed behavior are enhanced (CEN regions), while internally oriented or self-related cognition (DMN regions) is decreased (Menon and Uddin, 2010; Menon, 2015). This is well reflected in previous brain activation patterns where we observed relatively increased activation of frontal and parietal regions (CEN) during flow, while activation of the medial prefrontal cortex (DMN) mediating internally oriented cognition was relatively decreased during flow.

Therefore, to further investigate the role of the right anterior insula during flow, new data were acquired in a fresh sample of 41 healthy participants using blood oxygen level-dependent (BOLD) signal-based functional magnetic resonance imaging (fMRI) during our previously established flow paradigm (Ulrich et al., 2014, 2016b). This new data set has already been published in a recent study (Ulrich et al., 2022) where it was used to compute replication Bayes factors for the two central neural flow effects (U, invU). Among the various brain regions exhibiting an invU activation pattern, the right anterior insula came with decisive evidence for the replication effect (Ulrich et al., 2022).

While activation patterns are informative about the neural correlates of flow, they do not inform about how these changes in activation may arise. One approach to this issue is the analysis of task-based functional connectivity, which was employed here. As seed region, that part of the right anterior insula was used where the present sample has shown an invU activation pattern. For task-based functional connectivity inference, we employed the condition-weighted general linear model as implemented in the CONN toolbox (Whitfield-Gabrieli and Nieto-Castanon, 2012) which permits inferential comparison between task-related connectivity changes stemming from different conditions.

When comparing functional connectivity changes between flow and the boundary conditions of boredom and overload, we expected the anterior insula to be more strongly coupled during the flow condition with members of the CEN showing an invU-shaped activation pattern because the flow condition is of higher salience than the boundary conditions. Conversely, a U-shaped contrast of condition-related connectivity changes should demonstrate flow-related reduced coupling between the anterior insula and those aspects of the MPFC that had shown relatively decreased functional activation during the flow condition in our previous studies. If so, this observation would also constitute a critical observation against our previous computational model (Ulrich et al., 2016a) where downregulation of the MPFC during flow was shown to be effectively driven by relatively increased activation of the dorsal raphe nucleus during flow.

The salience network also includes three key subcortical structures—the amygdala, the ventral striatum (VS), and the substantia nigra/ventral tegmental area (SN/VTA) (Menon, 2015). For within-network functional couplings, we, therefore, predicted that particularly the amygdala should be coupled negatively with the right anterior insula, since downregulated amygdala activation has repeatedly been observed as another functional hallmark of flow. For the two other members of the salience network, that is, the VS and SN/VTA, no relation should exist at all. These structures code salience in the context of extrinsic motivation (e.g., Ulrich et al., 2018a) but so far have not been reported in the context of intrinsic motivation, for which the emergence of flow is almost prototypical (see above, autotelic activity).

## Materials and methods

### Participants

Forty-one healthy, male students from the local university, with an average age of 22.9 years (standard deviation, SD: 2.6 years), participated in the study. Participants did not report any psychiatric/neurological disorder or contraindications with regard to MRI. The study was in accordance with the Declaration of Helsinki and was approved by the Ethics Committee at Ulm University. Written informed consent was obtained before the experiment. Financial compensation was 40 EUR.

### Experimental task

As functional challenge, we used again the flow paradigm with mental arithmetic tasks of different difficulty levels to operationalize the three conditions of boredom, flow, and overload. The computational realization permits online adaptation of individual difficulty levels to reach the best possible balance between actual task affordances and individual skills and/or abilities. Furthermore, solving math calculations comes with very clear goal settings and clear feedback about whether the task could be solved. Therefore, the three necessary conditions for the emergence of flow are met in our flow paradigm (balance between the individual’s skills and task difficulty; clear goals; and immediate feedback about performance; see Csikszentmihalyi et al. (2014; p. 232).

On each trial, a math expression appeared in black font above an on-screen keyboard in the center of the screen. Stimuli were presented on white background on a 32-inches MR-compatible LCD screen (NordicNeuroLab AS, Bergen, Norway) at a resolution of 1024 × 768 pixels. The on-screen keyboard was controlled by an MRI-compatible trackball (Nata Technologies, Coquitlam, Canada). The computer algorithms generating and presenting the math calculations, analyzing the results, and performing the level adjustments were programmed in Scala version 2.9.01 and run in Java Runtime Environment version 6.0.05 on a standard PC with 64-bit Windows 7 Service Pack 1.

Participants were asked to sum the presented two or more numbers in their minds and to enter the result as accurately and as fast as possible using the on-screen keyboard. After a period of 10 s per each math calculation, or when participants had submitted the result, there was a break of 1 s during which the string “xxx + x” appeared on the screen. Calculations and intermittent breaks were presented until the block length of 30 s had elapsed. Depending on the specific experimental condition, task difficulty was either very low (boredom or “B” condition) or was challenging with dynamic adjustment of task difficulty to participants’ individual skill level (flow or “F” condition) or was overwhelming due to very high task difficulty (overload or “O” condition). In the boredom condition, subjects performed addition tasks involving always two numbers only, which were randomly drawn with the following restrictions: The first addend belonged to the interval of 100 to 109, the second addend belonged to the interval of 1–9, and the result was between 101 and 110. In the flow condition, task demands adapted automatically and continuously to participants’ level of skill, which had been estimated beforehand in a preparation phase (see below), and used as starting level. When the result was correct, task difficulty increased by one level, and when the result was incorrect, the level decreased by one level. Adjustment of task difficulty was achieved by two alternate mechanisms: In case the last addend had only one digit, the level increased by changing the last addend to a two-digit number in the next calculation. A further increase was accomplished by adding an additional one-digit number to the mathematical expression, and so on. Analogously, a decrease in task difficulty was achieved by the two mechanisms outlined above but in reverse order. In the overload condition, the starting level of task difficulty was set three levels higher than the starting level of the flow condition. The levels were adjusted in the manner described earlier but were not permitted to fall below the starting level. The experiment began with a block of a rest condition where subjects were asked to fixate on a black cross, centered on a white screen while avoiding unnecessary movements. Afterward, nine blocks per task condition, with intermitting rest blocks, were performed. Both task and rest blocks lasted 30 s. There were two predefined block sequences counterbalanced across participants: BFOBFBOFOFBOBFOBOFBFOFBOFOB and BOFBOBFOFOBFBOFBFOBOFOBFOFB (rest blocks between each of the B, F, and O conditions are not shown).

In the preparation phase, before scanning, participants underwent two practice blocks. The first block consisted of an extended version of the B condition, lasting 3 min, to make participants familiar with the trackball. After a short break of 15 s, a modified version of the flow condition was performed, aimed at determining participants’ individual level of skill: Starting at the lowest possible level, in the course of 4 min, task demands were automatically and continuously adapted to participants’ skills. The average task level pertaining to the last 25% of results was used as the starting level for the flow condition in the experiment.

As in our previous study (Ulrich et al., 2016b), the experimental manipulation described earlier was tested for effectiveness with subjective flow experience ratings and measurement of electrodermal activity as two separate non-imaging read-outs. Both measurements replicated (Ulrich et al., 2022) the same highly significant invU pattern for the present sample as in the previous study (Ulrich et al., 2016b). Therefore, experimental realizations and detailed results for both indices are not reported here see Ulrich et al. (2022).

### Magnetic resonance imaging data acquisition

Magnetic resonance imaging data were acquired on a 3 T MAGNETOM Prisma (Siemens AG, Erlangen, Germany) with a 64-channel head/neck coil. During the experiment, an echo-planar pulse sequence (EPI) was applied to measure the T2*-weighted BOLD signal. The following parameters were used: repetition time (TR) = 2000 ms, echo time (TE) = 33 ms, flip angle = 90°, PAT factor = 2 (GRAPPA mode), field of view (FOV) = 220 mm, matrix size = 90 × 90, number of slices: 32, slice orientation: transversal, acquisition: ascending, slice thickness: 3.0 mm, interslice gap = 1.0 mm, bandwidth = 2136 Hz/pixel, and voxel size = 2.44 × 2.44 × 4.00 mm. In about 27.5 min, 825 EPI volumes were acquired. To obtain a high-resolution T1-weighted structural image for later co-registration purposes, a magnetization-prepared rapid acquisition gradient echo sequence was employed: TR = 2300 ms, TE = 2.61 ms, inversion time = 1100 ms, bandwidth = 290 Hz/pixel, flip angle = 9°, FOV = 256 mm, matrix size = 256 × 256, voxel volume = 1 mm^3^, slice orientation: sagittal; PAT factor = 2 (GRAPPA mode), and scan time of about 5 min.

### Data analysis

#### Task-based activation

Preprocessing and statistical analyses were performed under MATLAB 2021a (MathWorks Inc., Natick, MA, USA) with Statistical Parametric Mapping (SPM12 r6225, Wellcome Department of Cognitive Neurology, London, UK) and the Computational Anatomy Toolbox 12 (CAT12.8.1 v1980^1^). Preprocessing started with the segmentation of individual T1 images using CAT12 with default settings (except for the voxel size of normalized images, set to 1 mm). This step produced bias-, noise-, and global intensity-corrected T1 images and tissue class images (gray matter, white matter, and cerebrospinal fluid) both in native and in normalized (Montreal Neurological Institute, MNI) space as well as forward deformation fields (native -> normalized). Functional MRI data were first slice time-corrected and spatially realigned. The resulting mean functional image was then co-registered to the individual, CAT12-processed T1 image in native space, and the co-registration matrix was applied to all other functional images. Afterward, the functional MR images were MNI-normalized by applying the forward deformation field from the initial CAT12 segmentation described earlier. Functional images were resampled to 2 × 2 × 2 mm and smoothed using a Gaussian kernel with 8 mm full width at half maximum.

A general linear model (GLM) was set up for each subject by specifying the onsets and durations (30 s) of the task blocks for B, F, and O as separate regressors. The spatial realignment parameters from each session were added to the design matrix as regressors of no interest. The resulting box-car functions were convolved with the canonical hemodynamic response function (HRF). To remove low-frequency scanner drifts, data were high-pass filtered with a frequency cutoff at 128 s, and an autoregression model of polynomial order 1 was used to account for temporally correlated residual errors.

A random effects group analysis was computed using a flexible factorial design with Condition as within-subject factor. A factor Subjects was added to the design matrix to account for the interindividual variance of estimated neural activation during each task condition against baseline. Two one-sided t-contrasts were computed to test for between-condition differences in form of polynomial trends of second order, that is, mirroring an invU and a U-shaped (U) activation pattern (see Introduction). For functional delineation of the right anterior insula as a seed region (see below), the invU effect was inferred at a significance level of *p* < 0.05, family-wise error rate (FWE)-corrected at the voxel level (whole-brain).

#### Task-based functional connectivity

Functional connectivity analyses were performed with the CONN functional connectivity toolbox 21a (Whitfield-Gabrieli and Nieto-Castanon, 2012; www.nitrc.org/projects/conn, RRID:SCR_009550). The following data from the preprocessing step above were passed to CONN: (1) the CAT12-normalized skull-stripped T1 images, (2) the CAT12-normalized tissue class images of the gray matter, white matter, and cerebrospinal fluid, (3) the CAT12-normalized smoothed (and unsmoothed) functional images, and (4) the realignment parameters (as first-level covariates). In addition, the onsets and durations of boredom, flow, overload, and rest blocks were specified.

The setup proceeded with definition of the seed region, from which the average time course was to be extracted. As stated earlier, the seed region had been functionally defined by the task-based activation analysis using the invU-contrast. At a level *p* < 0.05 (FWE-corrected), this resulted in a significant cluster (p(FWE) < 0.001) of 280 voxels located in the right anterior insula, which was masked with gray matter by the CONN toolbox.

In preparation for the denoising step, functional scans were screened for potential outliers (criteria: frame-wise displacements above 0.9 mm or changes in the global BOLD signal of the unsmoothed images above 5 standard deviations), and the resulting information was added as first-level covariates for later scrubbing. Actual denoising to remove potential confounding effects from the functional data was performed with CONN’s default denoising pipeline: Using the anatomical component-based noise correction method (aCompCor; Behzadi et al., 2007), noise extracted from the individual white matter and cerebrospinal fluid masks was estimated and regressed out from the BOLD signal. To account for the effects of motion, the realignment and scrubbing parameters were added as further confounds. The main task effects and potential linear trends were also removed from the signal. After the regression, data were high-pass filtered (cutoff: 0.008 Hz).

After the denoising step, seed-to-voxel functional connectivity was computed for the right insula as seed (CONN parameters: weighted GLM; HRF-weighting; bivariate regression). Resulting regression coefficient maps per condition and participant were transferred to a random effects group analysis implemented as a flexible factorial design with Condition (boredom, flow, overload) as a within-subject factor. A factor Subjects was added to the design matrix to account for the interindividual variance of estimated functional connectivity during each task condition.

Two one-sided t-contrasts were computed to test for between-condition differences in the form of polynomial trends of second order, that is, mirroring an invU and a U-shaped (U) functional connectivity pattern (see Introduction). Significant invU and U effects were inferred at a cluster level of *p* < 0.05, FWE-adjusted, that is, for each connectivity effect, at least 173 contiguously significant voxels at *p* < 0.001 were necessary to constitute a significant cluster at *p* < 0.05 FWE-corrected.

## Results

### The seed region

**FIGURE 1:**
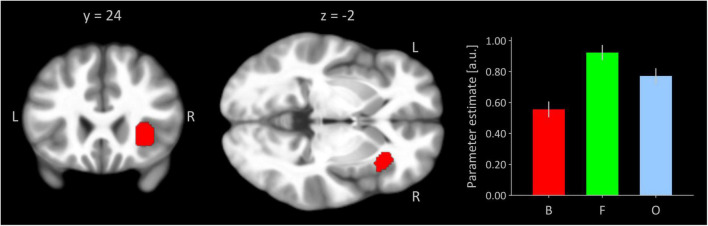
Right anterior insular seed region functionally defined by invU-shaped activation effect at a significance level of *p* < 0.05, whole-brain FWE-corrected. Cluster is overlaid on coronal and transversal sections obtained from participants’ MNI-normalized and averaged T1 images (*N* = 41) using MRIcroGL (Rorden and Brett, 2000). L, left; R, right. The bar graph depicts cluster-averaged estimated neural activation for conditions of boredom (B), flow (F), and overload (O); the error bar is the standard error of mean (*N* = 41).

Figure 1 shows the right insular seed region from the present task-based activation analysis inferred by a significant quadratic trend testing for an invU activation pattern. This trend is demonstrated by the inserted bar graph depicting cluster-averaged, estimated neural activation for conditions of boredom, flow, and overload for the seed. The cluster size was 280 significant voxels, and the peak voxel was located at MNI-coordinates [38, 24, −2] (z-value = 6.55, p(FWE) < 0.001).

### Task-based connectivity during flow vs. boundary conditions of boredom and overload

**FIGURE 2:**
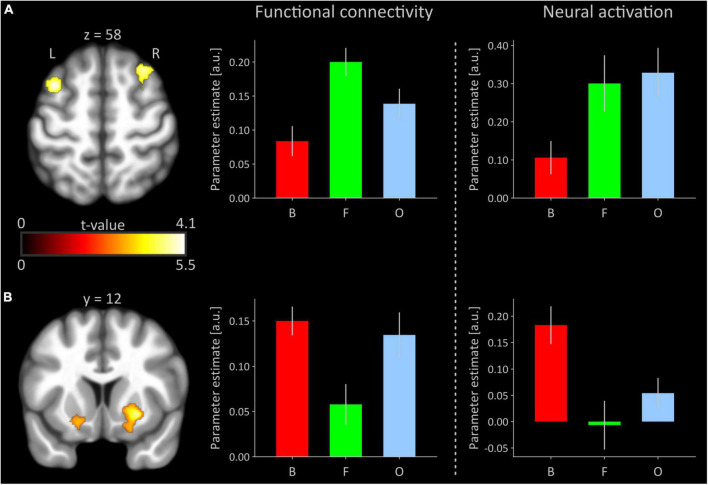
**(A)** Significant inverted U-shaped (invU) connectivity changes of prefrontal cortical regions with the right anterior insular seed. Clusters are overlaid on a transversal section obtained from participants’ MNI-normalized and averaged T1 images (*N* = 41) using MRIcroGL (Rorden and Brett, 2000). The bar graph in the middle depicts the cluster-averaged height of functional connectivity for the left dorsolateral prefrontal cluster (comprised of parts of the middle and inferior frontal gyri; cluster size: 462 voxels) for conditions of boredom (B), flow (F), and overload (O). The rightmost bar graph shows un-thresholded, estimated neural activation for each of the three experimental conditions, averaged across all voxels of the left dorsolateral prefrontal cortex that were significant in the functional connectivity analysis. **(B)** Significant U-shaped connectivity changes of ventral striatal regions with the right anterior insular seed. The bar graphs depict functional connectivity and neural activation, respectively, for the right putamen (250 voxels). The bar graph under neural activation shows again un-thresholded, estimated neural activation for each of the three conditions, averaged across all voxels of right putamen, significant in the functional connectivity analysis. Error bars refer to the standard error of mean. a.u., arbitrary unit; L, left; R, right. Averaged results for the right middle/superior frontal gyri and left putamen (see Table 1) in terms of functional connectivity and neural activation are presented in Supplementary Figure 1.

Significant polynomial trends of second order mirroring an invU-shaped connectivity pattern with the right anterior insular seed were observed for the left middle frontal and left inferior frontal gyrus, comprising aspects of the triangular and adjacent opercular part, and right middle frontal gyrus (see Figure 2A and Table 1, upper part). Inverting the contrast direction yielded a U-shaped connectivity pattern of left and right putamen (see Figure 2B and Table 1, lower part). Further brain regions, particularly the typical activation-downregulated aspects of MPFC and amygdala, were not observed, neither at the *a priori*-defined level of inference nor at an exploratory level of *p* < 0.01 without any cluster extent threshold.

**TABLE 1:** Summary of contrast-weighted task-based functional connectivity changes.

Brain region	Peak voxel (MNI space)			Peak voxel	Cluster size	Cluster
	x	y	z	z-value	k	p(FWE)
**Inverted U effect**						
Middle frontal gyrus (L)	−36	10	58	4.09	462	<0.001
Inferior frontal gyrus (L), opercular part	−52	20	34	3.74		
Inferior frontal gyrus (L), triangular part	−48	18	32	3.72		
Middle frontal gyrus (R)	30	20	56	3.92	180	0.042
Superior frontal gyrus (R)	26	20	48	3.59		
**U effect**						
Putamen (L)	−20	4	−12	5.38	183	0.040
Putamen (R)	26	12	−8	4.89	250	0.010

FWE, family-wise error rate-corrected; L, left; MNI, Montreal Neurological Institute; R, right.

## Discussion

In the present study, we investigated the role of the right anterior insula during the emergence of flow using a combined task-based activation and connectivity analysis in a sample of 41 healthy male participants. This research question was motivated by two previous observations: (1) Robust involvement of the right anterior insula during the flow condition of our experimental paradigm with stronger activations during flow compared to the boundary conditions of boredom and overload (e.g., Ulrich et al., 2022). (2) The previously described [for review, see Menon (2015) and Molnar-Szakacs and Uddin (2022)] role of the right anterior insula which, as a member of the salience network, is involved in the switching between the engagement of the central executive network (CEN) and disengagement of the default-mode network (DMN). The latter phenomena have repeatedly been observed in fMRI activation data of the flow condition relative to its boundary conditions (e.g., Ulrich et al., 2022).

For the task-based connectivity analysis, the right anterior insular seed was functionally derived from actual task-based activation data demonstrating the expected invU activation effect. Testing for the same effect direction (invU-shaped) in functional connectivity, we observed stronger functional connectivity with this seed during flow relative to the boundary conditions in the left and right dorsolateral prefrontal cortex, all regions representing members of the CEN (e.g., Seeley et al., 2007; Doucet et al., 2019). When inverting the effect direction (U-shaped), relatively reduced functional couplings during flow were found in the left and right putamen, where activation data showed the same effect direction (U-shaped).

Previous condition-weighted activation data indicate that the flow condition is relatively the most salient. This is supported by the repeatedly observed increased activation of brain regions serving externally oriented attention on the task and access to resources for goal-directed behavior (Ulrich et al., 2022), particularly under the flow condition. Also repeatedly shown was relatively reduced neural activation of the MPFC during flow in our previous studies (Ulrich et al., 2014, 2016a,b; Ulrich et al., 2018b) which reflects that internally oriented or self-related cognition is decreased, as another feature of high salience. The relatively increased activation of the right anterior insula during flow, therefore, aligns well with the presumed anterior insula’s role in detecting this between-condition difference in salience (Menon, 2015).

Given the right anterior insula’s role as a causal hub mediating engagement and disengagement of the CEN and DMN, respectively (Goulden et al., 2014; Menon, 2015; Chand and Dhamala, 2016; Krönke et al., 2020; Shaw et al., 2021; Molnar-Szakacs and Uddin, 2022), the relatively increased functional coupling during flow between this seed and the dorsolateral prefrontal regions is in good agreement with the predictions derived from this model. It is of note, however, that activation data of those regions did not exhibit the typical invU-shaped effect. Rather, the present activation pattern of functionally coupled prefrontal regions resembled much more a linearly increasing activation effect following the increased task difficulty of the three psychological conditions but not the flow-typical invU activation pattern. Furthermore, other members of the CEN, for example particularly the parietal cortices, could have been expected to show relatively increased functional coupling with the anterior insular seed. The absence of this effect indicates that the between-condition differences in functional coupling with the anterior insular seed were most likely not strong enough. It is of note here that the insula’s switching operation role has mostly been inferred from resting-state fMRI or task-based fMRI with only one condition against baseline or rest [see also Shaw et al. (2021)]. In the present study, however, three task conditions entered the task-based connectivity analysis, and all three conditions came with a functional challenge, which *per se* is associated with a specific salience, although at varying degrees.

This line of reasoning, however, may not account for the absence of relatively reduced (or even negative) functional coupling between the insular seed and those aspects of the MPFC, which have repeatedly shown clearly downregulated flow-associated activation. Hence, one might object that the invU-shaped connectivity pattern above cannot simply be inverted in a way that lower couplings between the insular seed and aspects of MPFC and amygdala would condition downregulation of activation there. On the contrary, for the ventral striatum as one of the within-salience network members, the predictions from the model hold and relatively reduced functional coupling with the right anterior insula was predictive of relatively reduced functional activation during flow. Insofar, the absence of U-shaped functional connectivity with the MPFC and amygdala is not necessarily an indication that the predictions from the model are wrong. Rather, the typically observed downregulated activation of the MPFC and amygdala is most likely driven by an input other than the anterior insula. An alternative path with the dorsal raphe nucleus as a source region has already been reported some time ago (Ulrich et al., 2016a).

The clearest picture of a U-shaped connectivity effect appeared for the left and right ventral striatum as a within-member of the salience network. Here, the contrast-weighted connectivity effect was in congruence with U-shaped activation data. This is interesting, as the ventral striatum is well known as one of the core regions of the so-called dopaminergic reward network (Haber and Knutson, 2010), processing prediction and outcome of extrinsic rewards like money, sex, or food. From the U-shaped activation/connectivity pattern, it appears that this system is almost inactivated during flow, and the markedly reduced functional couplings with the anterior insula suggest that this may happen under the influence of the right anterior insula. Due to the bi-directionality, functional couplings should not be interpreted as effective and the true mechanism behind this phenomenon must remain open. However, it is nevertheless intriguing that the most salient condition, flow, shows this dissociation with its boundary conditions in parts of the extrinsic reward system. It is therefore tempting to speculate that the intrinsically rewarding aspect of the flow condition perhaps even needs a relatively inactivated extrinsic reward system and that this relative inactivation is driven by the anterior insula as a detector of this specific form of salience.

One strong limitation of this and our previous work is that all studies so far had been performed in men-only samples. This restriction is due to the very first study, where we investigated the neural correlates of experimentally induced flow for the very first time and where we wanted to preclude any hormonal influences on neural activation measures that may come from hormonal changes during the menstrual cycle (Dietrich et al., 2001; Hausmann et al., 2002; Fernández et al., 2003). Since then, this selection bias was propagated due to methodological reasons. This limitation imposes the strong necessity to be healed by setting up a flow study with a women-only sample under hormonal control.

Another limitation is the focus on the right anterior insula. This focus was motivated by the mechanistic hypothesis for that region orchestrating engagement and disengagement of the CEN and DMN, respectively. However, other non-insular brain regions have also repeatedly revealed invU-shaped activation patterns very similar to that observed for the anterior insula. Future research should investigate if and how these regions contribute to the neural emergence of flow, particularly since previous studies (Spreng et al., 2013; Hellyer et al., 2014; Elton and Gao, 2015) already reported flexible coupling between both networks during cognitive control tasks. These between-network relationships during the flow paradigm were not the subject of the present study.

In conclusion, the present study investigated the role of the right anterior insula during the emergence of flow due to its repeatedly reported role as a causal out-flow hub orchestrating involvement of the CEN and DMN under conditions of higher salience. Task-based functional connectivity analysis showed indeed significant couplings with left and right aspects of dorsolateral prefrontal cortices as members of the CEN. However, the linearly increasing task-based activation pattern of these brain regions was not compatible with the typical flow-related invU activation pattern. A U-shaped functional connectivity pattern with the right anterior insula was evident only for the left and right putamen where the activation pattern was in congruence. However, for two other cortical structures for which U-shaped activation was repeatedly reported, the MPFC and the amygdala, no significant differential couplings with the right anterior insula were observed. The complete result pattern, therefore, suggests no substantial role for the right anterior insula in mediating the neural orchestration for the emergence of flow. The reduced coupling with aspects of the ventral striatum could be a hint for future research further disentangling the neural mechanisms behind the neural processing of extrinsic and intrinsic rewards, as the autotelic feature of flow is most representative of the latter.

## Data availability statement

The datasets presented in this article are not readily available because the authors do not have permission to share data. Requests to access the datasets should be directed to MU, martin.ulrich@uni-ulm.de.

## Ethics statement

The studies involving human participants were reviewed and approved by Ethics Committee at Ulm University, Germany. The participants provided their written informed consent to participate in this study.

## Author contributions

MU: conceptualization, methodology, data curation, formal analysis, software, visualization, writing—original draft, and reviewing and editing. FN: conceptualization, methodology, investigation, formal analysis, and writing—reviewing and editing. GG: conceptualization, methodology, formal analysis, project administration, supervision, writing—original draft, and reviewing and editing. All authors contributed to the article and approved the submitted version.

## Footnotes

1 http://www.neuro.uni-jena.de/cat; last accessed: 31 March 2022.
