# Supplementary material for: Role of the right anterior insula for the emergence of flow—A combined task-based fMRI activation and connectivity study
Source: Front Hum Neurosci. 2022 Dec 8;16:1067968. doi: 10.3389/fnhum.2022.1067968 (PMC9772033; doi:10.3389/fnhum.2022.1067968)
Supplement: Supplementary file 1 [file Data_Sheet_1.PDF]

Supplementary Material

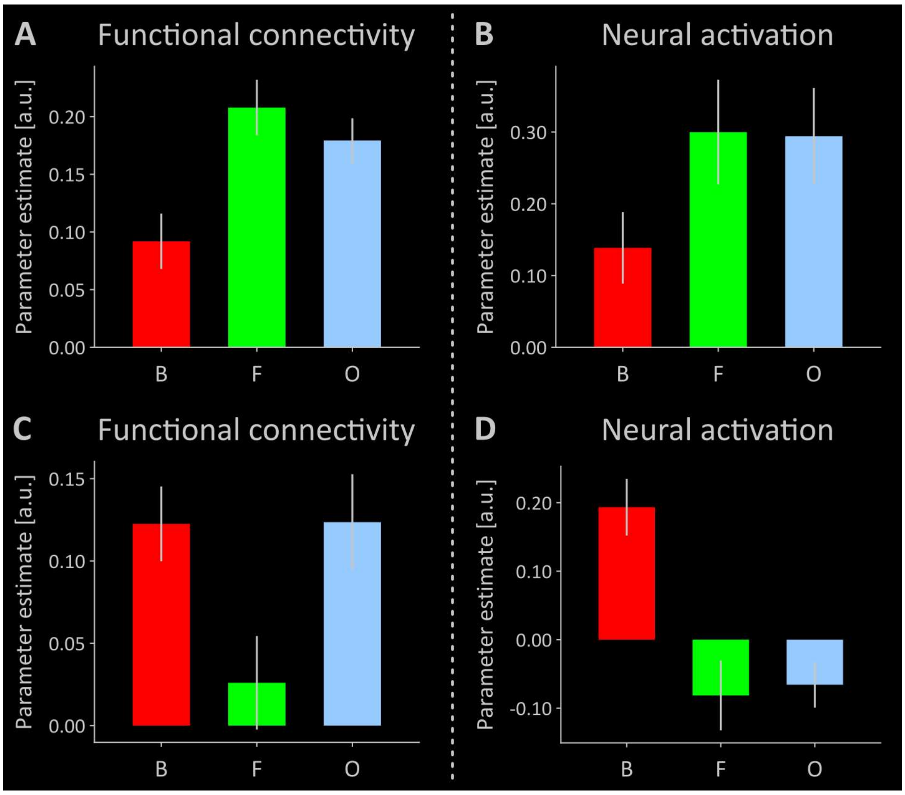

Supplementary Figure 1. (A) Graphical depiction of cluster-averaged height of functional connectivity for the right prefrontal cluster (180 voxels; Table 1 main article) for conditions boredom (B), flow (F), and overload (O). **(B)** Un-thresholded, estimated neural activation for each of the three experimental conditions, averaged across all voxels of the right prefrontal cortex cluster that were significant in the functional connectivity analysis. **(C)** Significant U-shaped connectivity changes of left putamen (183 voxels; Table 1 main article) with the right anterior insular seed. **(D)** Un-thresholded, estimated neural activation for each of the three conditions, averaged across all voxels of left putamen, significant in the functional connectivity analysis. Error bars refer to standard error of mean (N = 41). Abbreviation: a.u.: arbitrary unit.

Anatomical regions of selected resting state networks

Anatomical regions of selected resting state networks (RSN) as defined in the CAREN-Atlas (reproduced from: Doucet, G. E., Lee, W. H., and Frangou, S. (2019). Evaluation of the spatial variability in the major resting-state networks across human brain functional atlases. Hum. Brain Mapp. 40, 4577-4587. doi: 10.1002/hbm.24722; see also Doucet et al.'s Figure 5b of the main article for a graphical depiction, and their Supplementary Table S4):

- **Salience Network:**

  Anterior insula; dorsal anterior cingulate cortex; posterior regions in the supramarginal gyri and anterior precuneus

- **Central Executive Network:**

  Dorsolateral prefrontal cortex, superior parietal cortex; posterior inferior temporal cortex

- **Default Mode Network:**

  Medial prefrontal cortex/ventral anterior cingulate cortex, precuneus/posterior cingulate cortex, inferior frontal cortex, angular gyri, middle temporal cortex, parahippocampal gyri
